# Biochar and Cropping Systems Changed Soil Copper Speciation and Accumulation in Sweet Corn and Soybean

**DOI:** 10.3390/plants11182375

**Published:** 2022-09-12

**Authors:** Wenting Yang, Yuzhuo Pan, Xia Yu, Shihao Xiao, Weihu Wang, Meijuan Lu

**Affiliations:** 1 Key Laboratory of Crop Physiology, Ecology and Genetic Breeding, Jiangxi Agricultural University, Nanchang 330045, China; 2 College of Territorial Resources and Environment, Jiangxi Agricultural University, Nanchang 330045, China

**Keywords:** intercropping, biochar, accumulation, acid-soluble, oxidizable, straw

## Abstract

In order to explore the effects of biochar and cropping systems on soil copper (Cu) speciation and copper accumulation in sweet corn (*Zea mays* L. var. Rugosa Bonaf.) and soybean (*Glycine max* (L.) Merr.), three ratios of biochar (C0, 0%, C1, 2%, C2, 5% by mass ratio, (*w*/*w*)) and three cropping systems (monocropped sweet corn, MC; monocropped soybean, MS; sweet corn–soybean intercropping, CS) were studied under three Cu levels (Cu0, 0 mg·kg^−1^, Cu1, 200 mg·kg^−1^, and Cu2, 400 mg·kg^−1^) in a pot experiment. The following results were obtained: (1) Compared with C0, adding biochar (C1, C2) could significantly reduce the Cu concentration in sweet corn, and C2 significantly reduced the Cu concentration in soybean under Cu1 and Cu2; the Cu concentrations in sweet corn and soybeans under Cu1 were lower than 10 mg·kg^−1^. (2) Compared with MC or MS, C2 significantly reduced the Cu concentration (below the detection limit) in sweet corn and the Cu concentration (1.65 mg·kg^−1^) in soybean straw in CS under Cu1. The Cu concentration in sweet corn ears and soybean straw in CS under Cu2 also decreased significantly, reaching 1.84 and 10.36 mg·kg^−1^, respectively. (3) Compared with C0, C2 significantly reduced the soil acid-soluble Cu concentration under Cu1 and Cu2, but significantly increased soil oxidated Cu concentration. (4) Compared with MC, the concentration of soil acid-soluble Cu was significantly decreased in CSC1 under Cu2. Under Cu1, the concentrations of reducible Cu were significantly increased in CSC1 and CSC2, and the oxidizable Cu concentration was increased in CSC2. In conclusion, sweet corn–soybean intercropping combined with biochar 5% (*w*/*w*) is beneficial to reducing the concentration of acid-soluble Cu, and increases the concentration of oxidizable Cu in copper-contaminated soil. Under Cu1 (200 mg·kg^−1^), the Cu concentrations in sweet corn and soybean were lower than 10 mg·kg^−1^, which meets the national food safety standard of China. Under Cu2 (400 mg·kg^−1^), the Cu concentration in sweet corn was lower than 10 mg·kg^−1^, but it was higher than 10 mg·kg^−1^ in soybean.

## 1. Introduction

Copper (Cu) is an essential mineral nutrient for the proper growth and development of crops. It is potentially harmful when it occurs in excess of optimal concentrations [1]. Many studies have summarized the adverse effects of excess Cu on germination, growth, photosynthesis, and antioxidant response in agricultural crops [2]. There are anthropogenic and natural sources of Cu in soil, including copper mining, agrochemicals, fertilizers, pesticides, industrial waste disposal [3], etc. Swine manure has become a pollution source carrier because of its high Cu concentration [4]. A long-term (27 years) experiment reported that swine manure could increase soil Cu content and accumulation of Cu in rice grain [5].

Phytoremediation was one of the most sustainable techniques for soil heavy metal pollution remediation [6,7]. Intercropping was one of the effective phytoremediation methods for the remediation of Cu-polluted soil [8]. Moso bamboo–Sedum plumbizincicola intercropping could enhance the metal uptake capacity of Cu, zinc (Zn), and cadmium (Cd) in bamboo plantations [8]. *S. plumbizincicola*–*M. sativa* intercropping had high removal rates of Cu, the possible reason being that intercropping increased biomass and decreased soil pH [9].

In addition to phytoremediation, biochar addition also had a good remediation effect on soil heavy metal pollution [7,10,11,12]. Biochar could significantly reduce the accumulation of Cu in corn shoots, and soil available Cu content [13,14]. The immobilization effect of biochar on soil Cu is also obvious [15,16]. A study has shown that biochar combined with intercropping could reduce the absorption of cadmium and chromium in the shoots of *Machilus pauhoi* [17]. It is still unclear whether biochar combined with sweet corn–soybean intercropping could reduce the Cu uptake by the sweet corn and soybean and soil available Cu. This paper mainly studies the effects of biochar addition and sweet corn–soybean intercropping on crop Cu absorption and soil Cu speciation under soil Cu pollution, to clarify whether the intercropping system combined with biochar could reduce soil Cu availability and Cu uptake by sweet corn and soybean, and provide certain technical support for the sustainable development of agricultural production and ecological environment coordination.

## 2. Results

### 2.1. Crop Cu Content and Accumulation

Without biochar addition, soil Cu pollution seriously affected the normal growth of corn, especially in the corn–soybean intercropping system, the corn could not grow under Cu1 and Cu2. The highest Cu concentration was observed in Cu2C0MC. Biochar addition could alleviate the Cu stress in corn. Whether intercropped or monocropped, biochar addition (C1 and C2) drastically reduced the Cu concentration in corn straw and ears under Cu1 and Cu2 compared with C0. Compared with C1, adding more biochar (C2) could obviously reduce the Cu concentration, but significantly increased the Cu accumulation of sweet corn under Cu2 (Table 1).

Compared with monocropped corn, the intercropped corn could not grow under Cu1 and Cu2 without biochar. Under Cu1, the intercropping system significantly reduced the Cu concentration in corn ears with biochar addition (C1 and C2). Especially in C2, the Cu content of intercropped corn could not be detected by an atomic absorption spectrometer (AAS). Under Cu2, the intercropping system significantly reduced the Cu concentration and accumulation in corn ears, but obviously increased the Cu concentration and accumulation of corn straw with C2. The Cu concentrations in corn under Cu1 and Cu2 were all less than 10 mg·kg^−1^ with C2, which indicated that the Cu concentration in corn with C2 meets the national food safety standard of China, regardless of whether it is grown under Cu1 or Cu2.

Based on the present results (Table 2), soybean had better tolerance to Cu-toxicity than sweet corn. Without biochar, only the monocropped soybean could not grow under Cu2 stress. The highest Cu concentration in soybean was observed in Cu2C0CS.

In the monocropped soybean, compared with C0, biochar addition (C1 and C2) drastically reduced the Cu concentration in soybean under Cu1 and Cu2, but C2 significantly increased the Cu accumulation in soybean.

In the intercropped soybean, compared with C0, biochar addition (C1) drastically reduced the Cu concentration in soybean under Cu2, C2 drastically reduced the Cu concentration in soybean under Cu1 and Cu2; but C1 and C2 significantly increased Cu accumulation in soybean straw under Cu2, and Cu accumulation in soybean pod under Cu1, while C1 significantly increased Cu accumulation in soybean straw under Cu1 and soybean pod under Cu2.

Most of the Cu concentrations in soybean were more than 10 mg·kg^−1^, and only those in Cu1C2 treatment were less than 10 mg·kg^−1^. This indicated that soybean might uptake more Cu under Cu stress, and soybean had better Cu tolerance than sweet corn.

Compared with monocropped soybean, the intercropped soybean had notably higher Cu concentration in soybean straw under Cu1, and intercropped soybean could grow under Cu2 without biochar. This indicated that intercropping might alleviate Cu stress on soybean.

Compared with monocropped soybean, under Cu1, the intercropping system significantly reduced the Cu concentration in soybean straw with C1 and Cu accumulation in soybean straw with C2, but significantly increased Cu accumulation in soybean pod with C1 and C2. Under Cu2, the intercropping system significantly reduced the Cu accumulation in soybean with C2, but obviously increased the Cu accumulation in soybean straw with C1.

### 2.2. Soil Cu Concentration

The soil Cu concentrations had significant differences depending on Cu stresses. The cropping systems and biochar ratios had not affected the total soil Cu concentration (Table 3).

### 2.3. Soil Cu Speciation

**Figure 1:**
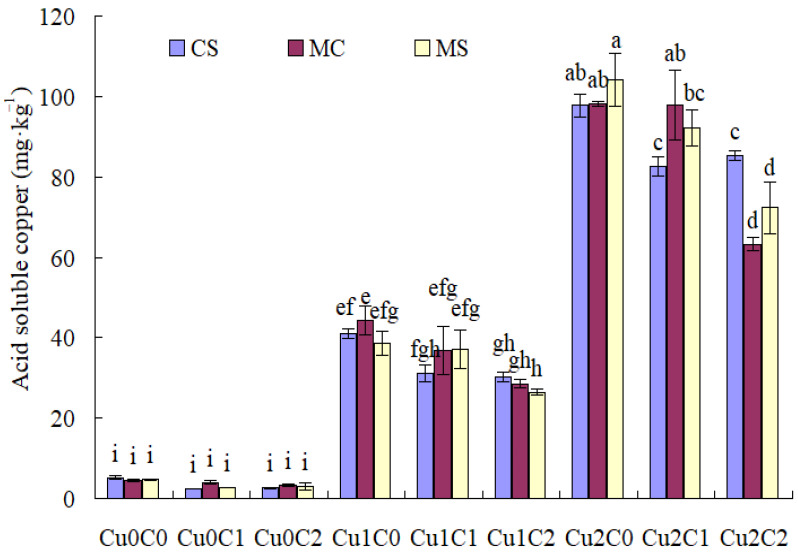
Soil acid-soluble copper concentration under different treatments. The different small letters above each column mean significantly different at *p* < 0.05.

With regard to soil acid-soluble Cu concentration (Figure 1), there was no difference with different cropping systems and biochar ratios under Cu0. Cu stresses (Cu1 and Cu2) drastically increased soil acid-soluble Cu concentration compared to Cu0. Compared with C0, whether intercropped or monocropped, C2 significantly reduced the soil acid-soluble Cu concentration under Cu1 and Cu2; C1 reduced the soil acid-soluble Cu concentration in CS and MS under Cu2.

Compared with monocropped sweet corn, the intercropping system significantly reduced soil acid-soluble Cu concentration with C1, but drastically increased it with C2 under Cu2. There was no significant difference with different cropping systems under Cu0 and Cu1 (Figure 1).

**Figure 2:**
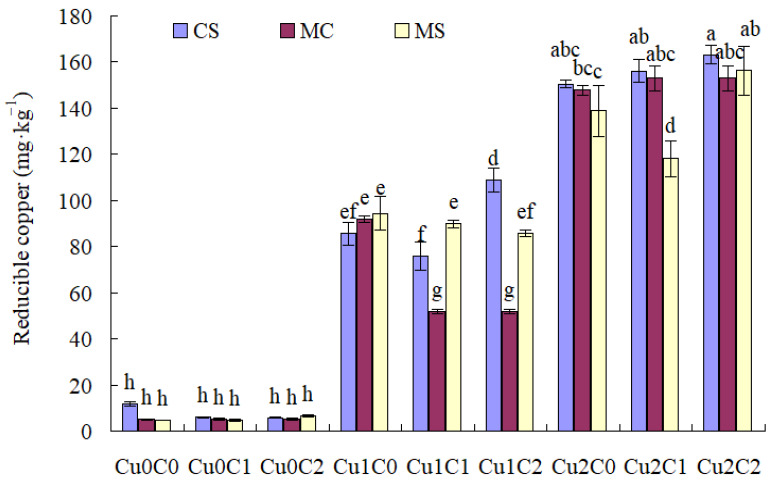
Soil reducible copper concentration under different treatments.The different small letters above each column mean significantly different at *p* < 0.05.

With regard to soil reducible Cu concentration (Figure 2), there was no difference with different cropping systems and biochar ratios under Cu0. Cu stresses (Cu1 and Cu2) drastically increased soil reducible Cu concentration compared to Cu0. Compared with C0, C1 and C2 significantly reduced the soil reducible Cu concentration in MC under Cu1, while C1 significantly reduced the soil reducible Cu concentration in MS under Cu2; C2 drastically increased soil reducible Cu concentration in CS under Cu1, and in MS under Cu2.

Compared with monocropped sweet corn, the intercropping system significantly reduced soil reducible Cu concentration with C1 and C2 under Cu1. Compared with monocropped soybean, the intercropping system significantly increased soil reducible Cu concentration with C1 under Cu2 (Figure 2).

**Figure 3:**
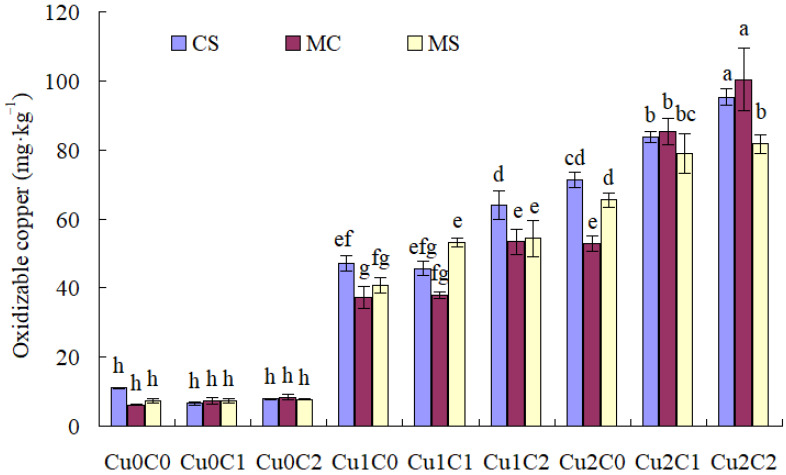
Soil oxidizable copper concentration under different treatments. The different small letters above each column mean significantly different at *p*<0.05.

With regard to soil oxidizable Cu concentration (Figure 3), there was no difference with different cropping systems and biochar ratios under Cu0. Cu stresses (Cu1 and Cu2) drastically increased soil oxidizable Cu concentration compared to Cu0.

Compared with C0, C1 and C2 significantly increased the soil oxidizable Cu concentration under Cu2, while C2 significantly increased the soil oxidizable Cu concentration under Cu1, and C1 significantly increased the soil oxidizable Cu concentration in MS under Cu1. Compared with C1, C2 significantly increased the soil oxidizable Cu concentration in CS and MC under Cu1 and Cu2.

Compared with monocropped sweet corn, the intercropping system significantly increased soil oxidizable Cu concentration with C0, C1 and C2 under Cu1, and with C0 under Cu2. Compared with monocropped soybean, the intercropping system significantly increased soil oxidizable Cu concentration with C2 under Cu1 and Cu2 (Figure 3).

**Figure 4:**
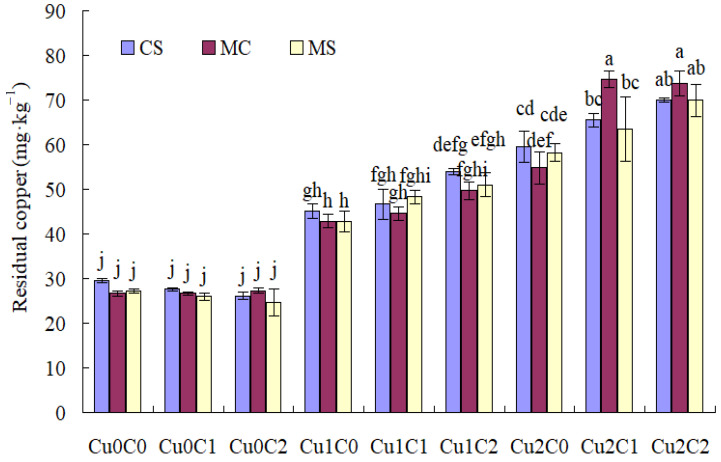
Soil residual copper concentration under different treatments. The different small letters above each column mean significantly different at *p* < 0.05.

With regard to soil residual Cu concentration (Figure 4), there was no difference with different cropping systems and biochar ratios under Cu0. Cu stresses (Cu1 and Cu2) drastically increased soil residual Cu concentration compared to Cu0.

Compared with C0, C2 significantly increased the soil residual Cu concentration under Cu2, and C1 significantly increased the soil residual Cu concentration in MC under Cu2. Compared with C1, C2 did not significantly affect the soil residual Cu concentration under Cu1 and Cu2.

Compared with monocropped sweet corn, the intercropping system significantly reduced soil residual Cu concentration with C1 under Cu2. Compared with monocropped soybean, the intercropping system did not affect soil residual Cu concentration under Cu1 and Cu2 (Figure 4).

## 3. Discussion

Compared with C0, adding biochar (C1, C2) could significantly reduce the Cu concentration in sweet corn, and C2 significantly reduced the Cu concentration in soybean under Cu stress. Speciation of Cu in soil solution was crucial to determining its toxicity to organisms [18]. Based on Figure 1, Figure 2, Figure 3 and Figure 4, biochar addition could reduce soil acid-soluble Cu and increase residual Cu, which reduced the bioavailability of copper and might inhibit copper absorption by crops. This might be one of the main reasons that biochar addition reduced the Cu concentration in sweet corn and soybean. Because biochar addition increased soil pH, available P, and soil enzymes [19,20], it indirectly promoted metal stabilization [21,22,23]. Soil acidification (pH 5.6 to pH 3.0) increased the mobile proportions of Cu [24]. Sugarcane biochar amendment reduced the availability of Cu in soil [25]. Studies have reported that wood and bamboo biochar application could decrease the concentration of Cu in maize shoot [26] and in soybean shoot [27], respectively. 

In the present results, biochar increased the edible safety of sweet corn by reducing acid-soluble Cu. The Cu concentrations in sweet corn and soybeans in C2 (5%, *w*/*w*) under Cu1 stress were both lower than 10 mg·kg^−1^. Plants absorb minimal quantities of Cu in the tissue range between 5 and 20 mg kg^−1^ dry matter in most species [28], but different crops have different tolerance to Cu [3]. In the present results, soybean had higher Cu tolerance than sweet corn, which might be because soybean cell suspensions could grow well in the high Cu concentrations [29]. The edible part of pak choi has been found to be safer for human consumption after biochar amendment [25]. Bamboo biochar addition (10%) also suppressed Cu uptake in soybean shoot [27].

The cropping system could control the heavy metal absorption by different crops. In the present results, compared with monoculture cropping, intercropping significantly reduced the Cu concentration in sweet corn and soybean straw in C2 under Cu1, and decreased the Cu concentration in sweet corn ear and soybean straw under Cu2. It might be that intercropping decreases Cu^+2^ in Cu-contaminated soils [30]. As shown in Figure 1, Figure 2, Figure 3 and Figure 4, compared with MC, the concentration of soil acid-soluble Cu was significantly decreased in CSC1 under Cu2. The CSC2 was also beneficial in increasing the oxidization of Cu concentration under Cu1. Agronomic practices might either alleviate or exacerbate Cu toxicity. The speciation of Cu in soil solution is crucial to determining its toxicity to organisms [18]. Intercropping enhances the metal uptake capacity of Cu, Zn, and Cd in bamboo plantations [8]. Compared with monocropping, Cd contents in the roots of cassava and seeds of peanut were significantly reduced by 20.00% and 31.67%, respectively [31].

## 4. Materials and Methods

### 4.1. Plant Material and Treatments

The experiment was conducted in Jiangxi Agricultural University Sci-tech Park, Nanchang China (28°46′ N, 115°50′ E). The soil is Ferralsols, collected from the 0–20 cm layer of the experimental field at the Sci-tech Park. The soil was air-dried, visible plant roots were removed, and sieved through a 2-mm sieve. The physical and chemical characteristics of the soil were as follows: pH 5.51 (1:2.5 distilled water), organic matter 24.70 g·kg^−^^1^, 1.21 total N available N 94.16 mg·kg^−^^1^, Olsen P 41.16 mg·kg^−^^1,^ and Available K 307.91 mg·kg^−^^1^.

**Figure 5:**
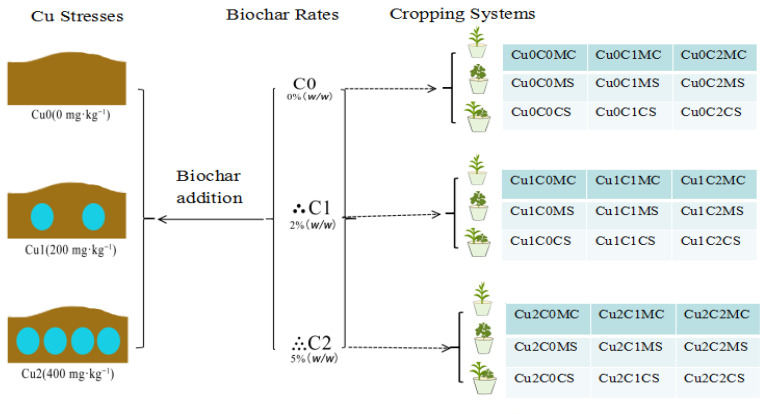
Experimental design and description of treatments. Note: 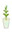 represents monocropped sweet corn (MC), 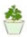 represents monocropped soybean (MS), 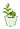 represents sweet corn–soybean intercropping (CS).

According to the farmland environmental quality evaluation national standard for edible agricultural products in China (HJ/T 332-2006), the limit of soil Cu concentration in edible crops land is 100 mg kg^−1^; the Cu concentrations in this experiment were set to 0 (Cu0), 200 (Cu1), and 400 mg·kg^−1^(Cu2). The remediation of Cu-polluted soil was performed using different cropping systems and biochar. The cropping systems included monocropped sweet corn (MC), monocropped soybean (MS), and sweet corn–soybean intercropping (CS); and the three biochar mass ratios were 0% (C0), 2% (C1), 5% (C2); the details are displayed in Figure 5. The experimental design was a randomized complete block with four replicates, the total pots were 108.

The corn cultivar ‘Ganketian 6’ was used. The soybean cultivar was ‘Taiwan 292’. The biochar was derived from the slow pyrolysis of peanut shells, produced at 500 °C by the Shangqiu Sanli New Energy Co., Ltd. (Shangqiu, China). The physicochemical properties of the peanut shell biochar were determined (Table 4).

The soil was mixed with biochar (0, 2, and 5%) and Cu (0, 200, and 400 mg·kg^−1^) by shovel until homogeneity was achieved. A 4.0 kg mixture of soil was filled into each plastic bowl two weeks before crop sowing. The plastic bowls used in the experiment were 220 mm in height, with upper and lower diameters of 180 mm and 150 mm, respectively. The bottom of the bowls had three holes (diameter 10 mm each) with two layers of nylon net (diameter 0.074 mm). The bowls were placed with a row spacing of 60 × 30 cm (60 cm between rows and 30 cm between plants within the rows) in the greenhouse. The soil was watered daily to field capacity (15%, *w*/*w*) after the corn and soybean were seeded.

Two corn seeds were sown into each plastic bowl (pot) in corn–soybean intercropping and monoculture corn, and four soybean seeds were sown per bowl in corn–soybean intercropping and monoculture soybean on 7 May 2019. One corn and two soybean seedlings remained in intercropping and monoculture soybean on 21 May. The corn and soybean were harvested on 1 August. The fertilizer rate in each pot was based on corn plant density in a local field (6.0 × 10^4^ plant·ha^−1^); N, P, and K were applied at 150, 45, and 135 kg·ha^−1^, respectively. Each pot had the same N, P, and K, except for the monoculture soybean without nitrogen application (Table 5). Basal fertilizer including urea 0.50 g·pot^−1^, potassium chloride 1.125 g·pot^−1^, and calcium superphosphate 6.25 g·pot^−1^ was applied before the crop was seeded. The first topdressing fertilizer, including urea 0.75 g·pot^−1^ and potassium chloride 1.125 g·pot^−1^, was applied when corn was at the corn jointing stage(V6) (20 June). The second topdressing fertilizer, including urea 1.25 g·pot^−1^ and potassium chloride 1. 5 g·pot^−1^, was applied at the corn tasseling stage(V14) (1 July).

### 4.2. Sampling and Measurements

Plants were sampled when the crops were harvested on 1 August. The corn and soybean straw (leaves and stem), and grain were separated, dried at 105 °C for 30 min, and then dried at 60 °C until to a constant weight. Dry weight was recorded. Dried plant samples were milled to 1 mm mesh and stored in small bags. The soil samples were air-dried (water content less than 5% (*w*/*w*)), visible plant roots were removed, ground, and passed through a 0.15 mm sieve. All plant and soil samples were digested with a mixture of HNO3-HCl (3:1) to measure the Cu concentrations by AAS (ICE 3000, Thermo Scientific, Waltham, MA, USA). The concentrations of available Cu speciation (acid-soluble, reducible, oxidizable, and residual fraction) in all soil samples were extracted by a modified BCR sequential extraction procedure described by Cuong et al. [32], and measured by AAS (ICE 3000, Thermo Scientific (Waltham), USA).

### 4.3. Statistical Analysis

The statistical significance of differences among treatments was tested with ANOVA (SPSS 13.0, SPSS Inc., Chicago, IL, USA), Duncan’s test was used to compare the means, and differences at *p* < 0.05 level were considered to be statistically significant.

## 5. Conclusions

The present results indicated that sweet corn–soybean intercropping combined with 5% (*w*/*w*) of biochar was beneficial to reducing the concentration of soil acid-soluble Cu, and increasing the concentration of oxidizable Cu in Cu contaminated soil. Under the 200 mg·kg^−1^ Cu stress, intercropping with biochar addition reduced Cu concentration and accumulation in sweet corn, but increased Cu accumulation in pods of soybean; the Cu concentrations in sweet corn and soybean were lower than 10 mg·kg^−1^, which could meet the national food safety standard of China. Under 400 mg·kg^−1^ Cu stress, intercropping with biochar addition increased the Cu concentration and accumulation in the straw of sweet corn, but decreased the Cu concentration and accumulation in ears of sweet corn; intercropping combined with 5% (*w*/*w*) of biochar reduced the Cu accumulation in soybean. Meanwhile, the Cu concentration in sweet corn was lower than 10 mg·kg^−1^, but the Cu concentration in soybean was higher than 10 mg·kg^−1^.

In conclusion, the sweet corn–soybean intercropping combined with 5% of biochar is a feasible planting pattern that yields safe production with heavy metal repair in low to moderate Cu-contaminated soil. It is recommended that the new planting pattern should be tested at the field scale in terms of its cost and practicability.

## Figures and Tables

**Table 1:** Cu content and accumulation of sweet corn.

Treatments		Concentration (mg·kg^−^^1^)		Accumulation (ug·plant^−^^1^)	
		Straw	Ear	Straw	Ear
Cu0C0	MC	1.87 ± 0.79 e	0	50.5 ± 19.91 c	0
	CS	0	0	0	0
Cu0C1	MC	0	0	0	0
	CS	0	0	0	0
Cu0C2	MC	0	0	0	0
	CS	0	0	0	0
Cu1C0	MC	20.19 ± 2.19 b	-	39.30 ± 2.14 c	-
	CS	-	-	-	-
Cu1C1	MC	3.03 ± 0.85 e	4.88 ± 1.01 b	47.80 ± 13.05 c	80.80 ± 11.15 b
	CS	1.58 ± 0.19 e	1.87 ± 0.18 c	26.90 ± 4.78 c	19.10 ± 0.82 c
Cu1C2	MC	2.92 ± 0.16 e	3.04 ± 0.40 c	157.80 ± 16.31 a	115.50 ± 24.79 b
	CS	0	0	0	0
Cu2C0	MC	25.17 ± 1.36 a	-	28.3 ± 0.71 c	-
	CS	-	-	-	-
Cu2C1	MC	14.41 ± 0.34 c	-	48.20 ± 3.64 c	-
	CS	17.35 ± 1.25 bc	-	23.70 ± 0.53 c	-
Cu2C2	MC	3.76 ± 0.46 e	7.67 ± 0.63 a	108.80 ± 14.7 b	171.60 ± 27.27 a
	CS	6.92 ± 0.36 d	1.84 ± 0.27 c	143.90 ± 2.09 a	32.40 ± 3.66 c

Note: The “0” means that the Cu content in the sweet corn is very low and it cannot be detected by AAS, and the “-” means the Cu concentration is too high, and the sweet corn cannot grow. The different small letters in the same column (straw or ear) mean significantly different at *p* < 0.05.

**Table 2:** Cu content and accumulation in soybean.

Treatments		Concentration (mg·kg^−^^1^)		Accumulation (ug·plant^−^^1^)	
		Straw	Pod	Straw	Pod
Cu0C0	MS	0	0	0	0
	CS	0	0	0	0
Cu0C1	MS	0	0	0	0
	CS	0	0	0	0
Cu0C2	MS	0	0	0	0
	CS	0	0	0	0
Cu1C0	MS	10.33 ± 1.65 de	6.16 ± 0.65 cd	49.00 ± 4.14 de	11.70 ± 0.68 d
	CS	17.57 ± 3.03 bc	9.41 ± 0.19 bc	52.20 ± 6.06 de	13.90 ± 3.53 d
Cu1C1	MS	21.74 ± 2.81 b	11.14 ± 2.77 b	30.90 ± 1.21 e	48.80 ± 3.33 d
	CS	12.99 ± 1.07 cd	9.26 ± 0.24 bc	120.60 ± 11.19 c	118.80 ± 7.96 bc
Cu1C2	MS	5.87 ± 0.37 ef	4.98 ± 0.49 d	122.20 ± 9.70 c	98.40 ± 12.59 c
	CS	1.65 ± 0.29 f	5.78 ± 0.45 d	44.90 ± 7.70 de	140.20 ± 9.10 b
Cu2C0	MS	-	-	-	-
	CS	34.47 ± 1.84 a	22.17 ± 1.26 a	76.00 ± 7.59 d	20.90 ± 1.22 d
Cu2C1	MS	18.23 ± 2.73 bc	23.35 ± 0.19 a	41.80 ± 3.09 e	39.20 ± 5.50 d
	CS	22.62 ± 2.07 b	11.45 ± 1.07 b	110.70 ± 13.33 c	32.70 ± 3.55 d
Cu2C2	MS	15.33 ± 0.41 cd	10.90 ± 1.07 b	301.80 ± 17.73 a	213.50 ± 33.05 a
	CS	10.36 ± 0.23 de	11.83 ± 0.53 b	162.60 ± 13.13 b	136.20 ± 14.69 bc

Note: The “0” means that the Cu concentration in the soybean is too low and it cannot be detected by AAS, and the “-” means the Cu concentration is too high, and the soybean cannot grow. The different small letters in the same column (straw or pod) mean significantly different at *p* < 0.05.

**Table 3:** Soil Cu concentration (mg·kg^−^^1^).

Treatments	MC	MS	CS
Cu0C0	55.54 ± 0.87 c	52.91 ± 0.54 c	54.27 ± 0.35 c
Cu0C1	53.31 ± 1.19 c	50.65 ± 0.64 c	53.43 ± 0.27 c
Cu0C2	53.80 ± 1.35 c	53.22 ± 2.74 c	55.67 ± 1.56 c
Cu1C0	258.18 ± 6.80 b	249.60 ± 2.87 b	254.05 ± 11.7 b
Cu1C1	245.46 ± 8.66 b	247.47 ± 3.34 b	246.62 ± 25.47 b
Cu1C2	241.25 ± 10.62 b	243.77 ± 12.97 b	263.09 ± 14.76 b
Cu2C0	415.57 ± 24.58 a	438.54 ± 28.54 a	415.08 ± 10.78 a
Cu2C1	420.00 ± 9.72 a	443.47 ± 7.45 a	413.57 ± 7.63 a
Cu2C2	424.78 ± 19.50 a	438.44 ± 23.87 a	451.49 ± 17.97 a

Note: The different small letters in the same column mean significantly different at *p* < 0.05.

**Table 4:** Properties of the biochar.

Properties	pH	Organic Matter (g·kg^−1^)	Total Nitrogen (g·kg^−1^)	Total Phosphorus (g·kg^−1^)	Total Potassium (g·kg^−1^)
	9.16	323.76	10.52	44.73	15.51

**Table 5:** Fertilizer rates.

Treatments	Monoculture Corn	Corn–Soybean Intercropping	Monoculture Soybean
Nitrogen (kg·ha^−1^)	150	150	0
Nitrogen (g·pot^−1^)	2.50	2.50	0
Urea (g·pot^−1^)	5.43	5.43	0
Potassium chloride (g·pot^−1^)	3.75	3.75	3.75
Calcium superphosphate (g·pot^−1^)	6.25	6.25	6.25

## Data Availability

The datasets used during the current study are available from Meijuan Lu on reasonable request.
